# Exploring alternative fuel solutions: lemon grass oil biodiesel blend with dibutyl ether additive for VCR diesel engines - an experimental analysis

**DOI:** 10.1038/s41598-024-70491-7

**Published:** 2024-08-31

**Authors:** Prabhu Paramasivam, Arun Balasubramanian, Adekunle Akanni Adeleke, Peter Pelumi Ikubanni, Sandeep Kumar, Chander Prakash, Rahul Kumar

**Affiliations:** 1 Department of Research and Innovation, Saveetha School of Engineering, SIMATS, Chennai, Tamilnadu 602105 India; 2 Department of Mechanical Engineering, College of Engineering and Technology, Mattu University, Mettu, 318 Ethiopia; 3 Department of Mechanical Engineering, Sethu Institute of Technology, Pulloor, Kariapatti, Tamilnadu 626115 India; 4 Department of Mechanical Engineering, Nile University of Nigeria, Abuja, 900001 Nigeria; 5 Department of Mechatronics Engineering, Bowen University, Iwo, Osun State Nigeria; 6 Division of Research and Development, Lovely Professional University, Phagwara, 144411 Punjab India; 7 University Centre for Research and Development, Chandigarh University, Mohali, Punjab 140413 India; 8 KKR and KSR Institute of Technology and Sciences, Guntur, Andhra Pradesh, 522017 India

**Keywords:** Sustainability, Biodiesel, Variable compression ratio, Engine performance, Fuel additives, Environmental impact, Mechanical engineering

## Abstract

There has been an intense surge in interest in the search for alternative sources of petroleum fuels in the modern world as a result of the inflation of fuel prices and the historic supply gap. When compared to petroleum fuels, biodiesel is becoming an increasingly valuable option due to the fact that it produces less emissions and provides the almost same amount of energy. In point of fact, the prime aim of this work is to explore the possibility of utilizing biodiesel derived from lemongrass oil and including dibutyl ether as an additive for the test diesel engine operating on varied compression ratios. The findings showed that the best operating settings are a 17.5 compression ratio with a blend of 30% biodiesel and 70% diesel fuel. At greater loads, brake thermal efficiency is lower than that of diesel engines. Lower loads result in lower specific fuel usage. Mechanical efficiency at higher loads is highest in the B30 blend, but emission metrics such as CO, CO_2_, HC, and NOx were reduced with the inclusion of an additive, though HC rose with higher loads of lemongrass oil biodiesel blends. When compared to the B30 biodiesel blend with various composition additives, the B30 + 4% additive has the highest efficiency at the fourth load in terms of both brake power and mechanical efficiency.

## Introduction

The usage of biodiesel has the potential to significantly contribute to sustainable development goal 7 (SDG 7), which seeks to provide all people with economical, sustainable, and modern energy^1^. Biodiesel, being an ecologically beneficial fuel, has the potential to improve energy availability in isolated and rural regions, enhance energy independence, and minimize reliance on imported fossil fuels^2^. Biodiesel, derived from renewable feedstocks, decreases reliance on finite fossil fuel supplies, leading to a more environmentally friendly and sustainable energy mix^3^. Because of its lower greenhouse gas emissions, it helps to combat climate change and supports SDG 13 on climate action. Biodiesel improves air quality by generating lower amounts of pollutants, thereby helping achieve SDG 3 on good health and well-being^4^. In addition, biodiesel production and utilization are in line with SDG 12 on responsible consumption and production. Furthermore, biodiesel operations have the potential to boost local economies, generate job opportunities, and contribute to SDG 8 on decent work and economic growth^5^. SDG 9 on innovation, industry, and infrastructure may be promoted through fostering the transfer of knowledge, building capacity, and research in the biodiesel area. Overall, the use of biodiesel promotes sustainable energy practices, decreases emissions, improves air quality, and contributes to sustainable development, making it an important instrument in reaching SDG 7 and progressing towards a more sustainable future for all^6^.

The diesel engine has become well-known for its contributions to society. Its main draws are its tough structure, ease of support, and simplicity of use. Because of the higher brake thermal efficiency (BTE) and lower fuel consumption, it has become an established machine in the transportation and agriculture sectors^7^. Excellent compression ratios, leaner fuel–air mixes, and little pumping losses as a result of throttle absence all contribute to high thermal efficiency^8^. However due to the rapid depletion of fossil fuel, rising costs, erratic supplies, rising petroleum demand, and, most crucially, strict emission regulations, experts are now seriously looking for alternatives to diesel fuel^9^. Therefore, it is crucial to create a long-term plan for the development of alternative energy sources that are balanced and makes the best use of the resources available in terms of land and labor. It is crucial to look into the possibilities of replacing diesel with an alternative fuel that can be generated on a large scale in the country for commercial use^10^. As a result, efforts are being made globally to identify suitable replacement fuel for diesel engines. Due to their greater heat efficiency, lower carbon monoxide emissions (CO), higher load capacity, and unburned hydrocarbons (UHC), diesel engines are significantly more cost-effective^11^. Diesel fuel consumption has increased exponentially as a result, and demand has recently grown. Rapid exhaustion of fossil fuel reserves owing to increased diesel fuel utilization increases demand and diesel market prices^12^. In-depth research is being done in the study community to develop an alternative fuel that can take the place of diesel fuel to meet supply and demand^13^. Soot emissions and nitrogen oxide (NOx) emissions from diesel engines are also very concerning. Lowering NOx and soot simultaneously is a challenging task for academics since they both have trade-off attributes^14^.

Suresh et al.^15^ studied the application of green synthesized carbon nanotubes as nano additives in ternary fuel mixes for a CRDi diesel engine (diesel (60%), ethanol (20%), and algal biodiesel (20%). When the carbon nanotube concentration in the fuel blend was increased from 25 to 100 ppm, engine performance improved, with a 9% increase in BTE, an 11.76% reduction in BSFC, and a 12.79% decrease in BSEC compared to diesel. In contrast to diesel and other blends, nanotubes blended biodiesel blend helped in emission reduction of HC by half, CO almost 14%, a reduction of 13.42% in NOx. Joshi et al.^16^ tested algae biofuel combined with diethyl ether (DEE) in a mono-cylinder, direct-injection CI engine. When compared to diesel, the inclusion of DEE resulted in a 7.2% gain in maximum BTE and a 6.3% reduction in minimum BSFC, with reductions in HC (12%), CO (19%), and a little rise in NOx (3%) emissions. Balasubramanian et al.^17^ explored the influences of CR on a 5.5-kW diesel engine running on B100 biodiesel. The engine's thermal efficiency improved as the CR was increased, with a 9.5% and 4.63% gain in brake thermal efficiency at full load for CRs of 21:1 compared to the original CR of 19:1. However, at higher CRs, NOx emissions showed elevated results. However, higher CRs increased NOx emissions, which might be mitigated by delaying injection timing. Overall, these studies show that using new fuel blends and engine changes can improve performance and reduce emissions in diesel engines. Yildirim et al.^18^ set out to assess the vibroacoustic characteristics of biodiesel made from used cooking oils with petroleum-based diesel fuel. The tests were carried out on a diesel engine with a 6 L cylinder volume, with an emphasis on altering engine speed & load. At high engine speeds, a vibration study using root mean square and coherence methods found that the vibration amplitude of B100 (100% biodiesel) was slightly greater than pure petroleum-based diesel fuel (PBDF). At 1500 rpm, the maximum vibration amplitude of B100 was found to be 8.5% more than that of PBDF. Coherence investigation revealed that engine sound increased with engine speed, reaching a maximum noise level of 94.9 dB with B100 at 2000 rpm. Another investigation by Rao et al.^19^ examined the effect of carbon nanotubes (CNTs) on the efficiency of a diesel engine utilizing a biodiesel-diesel blend (Y20). The addition of optimal quantities of CNT nano additions resulted in considerable increases in a variety of performance and emission metrics, including BSFC, BTE, CO_2_, CO, HC, and NOx. CNTs also increased cylinder pressure, heat release rate, pressure rise rate, ignition delay, and combustion duration. Overall, the inclusion of CNTs was found to improve performance and lower emissions in the Y20 blend, demonstrating CNTs' potential as useful fuel additives.

For the literature analysis, it could be seen that there has been not much research into the emissions and performance features of fuel mixes containing diesel, lemongrass biodiesel, and dibutyl ether. Moreover, the impact of different engine loads and compression ratios on the combustion and emission characteristics associated with these fuel blends have not been thoroughly investigated. In addition, the synergistic effects of mixing lemongrass biodiesel with dibutyl ether as diesel fuel additives have not been properly studied. Thus, the prime objective is to explore the emissions and performance features of test blends comprising diesel, lemongrass biodiesel, and dibutyl ether under various engine load circumstances and compression ratios. The study aims to fill current gaps in the literature by:

- Evaluating the impact of different blend compositions on the performance of engine and efficiency.
- Examining the effect of various blend ratios on exhaust emissions like CO, HC, and NOx.
- The effects of combining lemongrass biodiesel with dibutyl ether additives on combustion effectiveness, ignition characteristics, and engine sound are being studied.
- The effects of combining lemongrass biodiesel with dibutyl ether additives on combustion effectiveness, and ignition characteristics are being studied.
- Offering insights into ideal blend compositions that could enhance engine performance while lowering emissions, hence contributing to the development of environmentally friendly and sustainable fuel solutions.

By addressing these objectives, the research aims to add to existing knowledge on the use of lemongrass biodiesel and dibutyl ether as additives in diesel fuel, providing valuable insights for engine optimization and the development of more efficient and cleaner fuel blends.

## Material and methods

### Lemongrass biodiesel

Lemongrass biodiesel is produced from citronella grass (*Cymbopogon Nardus*). Lemongrass is a special plant with fragrant and therapeutic characteristics. This Poaceae family perennial grass is native to tropical climates. Citronella grass grows to a height of one to two meters owing to its tall and thin stems^20^. The leaves are thin and long, and when crushed, they emit a strong lemon-like aroma, hence the name. Lemongrass oil was extracted by using steam distillation^21^. The process involves heating lemongrass in a chamber with steam from an external boiler. The high temperature causes the oil to be removed from the lemongrass. The biodiesel was prepared using well well-established transesterification process. The main properties of the test fuel are depicted in Table 1.

**Table 1:** Properties of lemongrass oil biodiesel.

Properties	Diesel	Lemongrass oil biodiesel
Density (kg/m^3^)	832	894
Viscosity (mm^2^/s)	2.71	3.8
Lower calorific value (MJ/kg)	42.4	37.1
Cetane number	42	50
Flashpoint (°C)	69	68

### Dibutyl ether

Dibutyl ether is an additive that contains oxygen. It is a flammable, colorless liquid with the chemical formula C_8_H_18_O. Because there is oxygen present, the fuel burns more efficiently, resulting in good combustion. Dibutyl ether, abbreviated as DBE, is a multifunctional chemical molecule that plays a significant part as a diesel fuel additive. It is an ether that is formed up of two butyl groups connected to an oxygen atom. DBE is a colorless, transparent liquid with a distinct odor. DBE has multiple significant benefits as a diesel additive^22^. For example, it improves the combustion qualities of diesel fuel, resulting in improved fuel economy and lower pollutants. DBE enhances the brake thermal efficiency (BTE) of diesel engines by helping in the improvement of combustion, resulting in greater energy efficiency and lower specific fuel consumption. DBE helps to maintain optimal engine efficiency, avoid injector fouling, and reduce fuel filter blockages by keeping the fuel system clean, therefore increasing the engine's lifespan^22^. Furthermore, DBE improves the ignition quality of diesel fuel by acting as a cetane number improver. A greater cetane number enhances combustion, resulting in smoother engine running, less ignition delay, and lower amounts of unburned hydrocarbons and particulate matter in the exhaust. This significantly reduces hazardous pollutants like CO, NOx, and HC, assisting in satisfying severe environmental standards and improving air quality. DBE has high stability, compatibility, and miscibility with diesel fuel, in addition to its combustion-enhancing qualities^23^. It easily mixes with diesel, guaranteeing a uniform mixture without requiring sophisticated or costly adjustments to the current fuel infrastructure. As a result, DBE is a viable and affordable diesel additive for both traditional and modern diesel engines^24^. The properties of DEE are listed in Table 2.

**Table 2:** Properties of Dibutyl ether.

Properties	Dibutyl ether
Density (kg/m^3^)	770
Viscosity (mm^2^/s)	0.855
Calorific value (MJ/kg)	38.3
Cetane number	90
Flashpoint (°C)	25
Oxygen content (%)	12.25

### Transesterification process

Biodiesel shares many of the same characteristics as diesel fuel. The esterification process improves the vegetable oil's density, viscosity, cetane number, calorific value, atomization and vaporization rate, molecular weight, and fuel spray penetration range^25^. In the biodiesel production process, a 500 mL conical flask was filled with Lemongrass oil and heated for 15 min (55 °C). A solution was made by combining a pellet of sodium hydroxide weighing 4 g with 60 ml of ethanol in a beaker that held 250 ml. Following a sustained period of intense agitation of the solution, the sodium hydroxide pellet was completely dissolved. With the addition of the sodium ethoxide solution, the lemongrass oil was prepared. To get the temperature of the blended solution up to sixty degrees Celsius, it was heated in a water bath for sixty to ninety minutes. After that, the mixture was allowed to settle in a burette for twenty-four hours. The two components that make up the bottom layer are glycerol and soap, while the top layer is composed of crude biodiesel. The next step is to separate the crude biodiesel and glycerol into their respective containers. In the subsequent step, water was utilized to remove any traces of soap and glycerol that were still present in the crude biodiesel. Up to the point where the biodiesel could be seen beneath the clear water in the burette/separating funnel, this process was carried out. After washing the sample, it was placed on a hot plate to dry, and any water that was still present in the biodiesel was extracted to eliminate it. After the collection was complete, the volume of pure biodiesel was meticulously measured and documented.

### Ethics approval and consent to participate

#### Plant guidelines

The Lemongrass plants used in our research were cultivated according to locally established agricultural practices. We confirm that no wild plant materials were utilized in our study. This study including plant material complied with relevant institutional, national, and international guidelines and legislation.

#### Permission to collect the plants/plant parts

The Lemongrass plants utilized in our study were sourced from a local nursery that specializes in herb cultivation. The established parameters were followed when gathering the cultivated lemongrass plants.

#### Source of the plant used in your study

The Lemongrass plants utilized in our research were sourced from a local reputable nursery specializing in herb cultivation. We ensured that the nursery followed proper agricultural practices and adhered to any relevant regulations governing plant cultivation and distribution. Additionally, we maintained records of the specific nursery from which the Lemongrass plants were obtained for traceability purposes.

## Experimental setup

**Figure 1:**
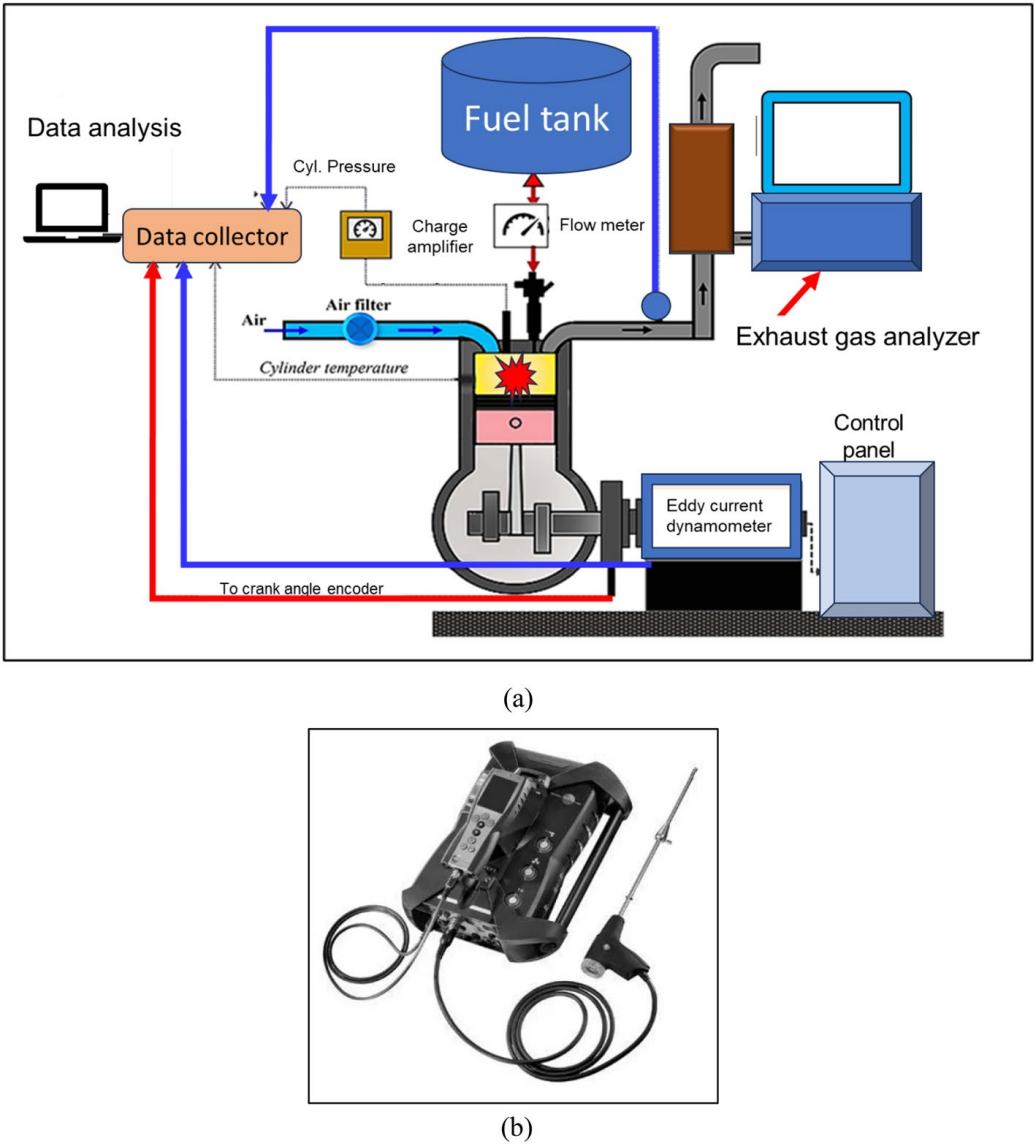
Test set up (**a**) engine (**b**) emission analyzer.

Figure 1a presents a schematic overview of the experimental setup, and Table 3 provides information regarding the parameters of the Kirloskar make diesel engine. The engine was allowed to run at a low load for twenty minutes in order to warm up. It was done so that temperature stabilization of engine oil and cooling takes place. The engine load was increased gradually while keeping the engine speed of 1500 revolutions per minute. At this fixed engine speed, other parameters were varied like compression ratio (CR), load, and blending ratio. The load on the engine was varied using an eddy current-based dynamometer. The engine has an in-built provision for varying the CR. The different blending ratios of test fuel samples were prepared using the ultra-sonication-based mixer. The data for the study was gathered through the utilization of an electronic acquisition system supplied by Apex Innovation, India. The engine is outfitted with a Kistler make pressure sensor for measuring the cylinder pressure. There are five thermocouples distributed over five separate sites on the engine. The data-collecting device is linked to all of the sensors and displays real-time data on the personal computer. The emission analyzer is Testo make and was used to measure different emissions such as CO, unburned HC, and NOx. The emission analyzer is shown in Fig. 1b. Further details on the engine and test setup are listed in Table 3.

**Table 3:** Specifications of Kirloskar diesel engine.

Name	Description
Rated power (kW)	3.5
Rated speed (rpm)	1500 ± 50
Bore diameter (mm)	87.5
Cooling type	Water based
Stroke (mm)	110
Compression ratio	Variable type from 16:1to18:1
Bore (mm)	85
Piezo based pressure sensor	Model – 6058A, Range – 0 to 250 bar, Sensitivity – of − 17pC/bar, Acceleration sensitivity: < 0.0005 bar/g, Operating temperature range: − 20 to 400 °C
Crank angle encoder	Type: optical Resolution of crank angle signal: 720 × 0.5° Dynamic accuracy at 10 000 1/min signal delay: 0.02 °CA Speed range: 0 to 20 000 rpm Operating temperature range: − 30 to 115 °C

In experimental studies, it is important to minimize the error and uncertainties^26^. Each set of engine tests was conducted thrice to reduce the uncertainty and errors. By choosing the appropriate instruments, their condition, calibration, observation process, test procedure, and planning, it is possible to decrease the number of errors and uncertainties that occur during the performance of experiments. However, it is impossible to eliminate them^27^. The uncertainties and errors may creep in due to ambient conditions, manufacturing defects, calibration errors, etc. The uncertainty can be estimated as^28^:

1 $$R=R \left({Z}_{1}, {Z}_{2}, {Z}_{3}, \dots , {Z}_{n} \right).$$

The partial derivative is given as^29^:

2 $$\frac{{U}_{R}}{R}= \sqrt{{\left(\frac{{Z}_{n}\partial R}{R{\partial Z}_{n}}\right)}^{2}\frac{{{U}^{2}}_{{Z}_{n}}}{{{Z}^{2}}_{n}}.}$$

Herein, R denotes an independent variable, $\frac{\partial R}{{\partial Z}_{1}}$ is the estimated sensitivity of individual variables. The accuracy, range, and uncertainties are listed in Table 4.

**Table 4:** Accuracy and uncertainties in measurement.

Parameter	Range	Accuracy	Uncertainty, %
Pressure sensor	0–250 bar	±1 bar	± 0.5
Temperature	0–1200 °C	± 1 °C	± 1
Fuel supply rate	0–100 mL	± 1 mL	± 0.5
Exhaust emission	HC	0–40,000 ppm	± 0.1
	NOx	0–3000 ppm	± 0.5
	CO	0–50 Vol. %	0.25
	CO_2_	0–50 Vol. %	0.3

## Results and discussion

The experimental results that were acquired from the studies are given an in-depth analysis in this part. In the course of the trials, the performance and emissions characteristics of fuel blends that contained diesel, biodiesel (varying from 0 to 30%), and dibutyl ether were analyzed. The engine load and compression ratio settings were changed during the course of the studies. There is a greater knowledge of the effect that certain fuel blend compositions have on engine performance, including characteristics such as SFC, BTE, mechanical efficiency, and other emission metrics, thanks to this section's in-depth examination and interpretation of the data that was acquired. During the course of the conversation, we looked into the patterns, correlations, and amazing outcomes that were discovered, providing an understanding of the potential benefits and drawbacks associated with the different fuel mix compositions. The data was additionally compared to the base diesel fuel to see whether there were any benefits or drawbacks in terms of engine performance and emissions caused by the various blending levels of test samples.

### Effect of compression ratios

**Figure 2:**
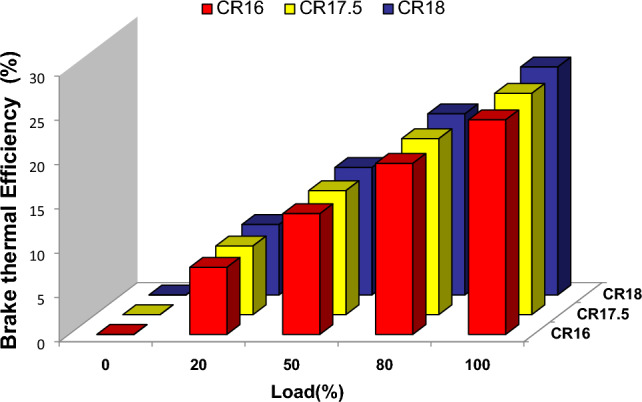
Load vs BTE at the different compression ratios.

When it comes to evaluating an engine's performance and fuel consumption, the connection between compression ratios and the thermal efficiency of the engine is of the utmost importance. Under pure diesel circumstances, Fig. 2 illustrates the load vs brake thermal efficiency of four different compression ratios (CR16, CR17.5, and CR18) at five distinct load points: zero, twenty percent, fifty percent, eighty percent, and one hundred percent. According to the findings of the tests, higher compression ratios are associated with improved thermal efficiency of the brakes, which ultimately leads to lower consumption of test fuel^30^. The findings of this investigation are in agreement with the fundamental laws of thermodynamics, which suggest that higher compression ratios result in improved combustion efficiency and energy conversion within the engine^31^.

The study also highlighted the effect of varying the CRs on BTE. The analysis discovered that increasing the cut-off ratio reduced brake thermal efficiency. This fact implies that at greater cut-off ratios, the engine's expansion mechanism became less successful, resulting in a loss in total thermal efficiency^32^. When the performance of the compression ratios CR16, CR17.5, and CR18 were examined, it was revealed that CR17.5 gave the best combination. This means that a compression ratio of CR17.5 achieves a reasonable balance of boosting brake heat efficiency while reducing the drawbacks associated with exceptionally high compression ratios^33^. These findings have significant implications for engine design and optimization because they highlight the need to select an appropriate compression ratio for best performance in terms of BTE as well as BSFC. Similar results are reported in the case of biodiesel used to power the diesel engine^34^.

**Figure 3:**
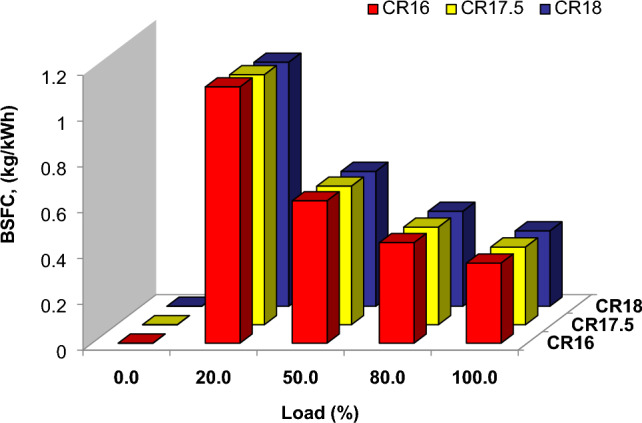
Load vs BSFC of different compression ratio.

Figure 3 demonstrates the relationship between load and brake-specific fuel consumption (BSFC) using pure diesel fuel at five load levels (0, 20, 50, 80, and 100%) for different compression ratios (CR16, CR17.5, and CR18). The testing findings reveal that BSFC reduces at higher engine load across all tested fuels. This is because enhanced Brake Thermal Efficiency (BTE) at higher loads exceeds the equal rise in fuel consumption^35^. When the performance of the three compression ratios, CR16, CR17.5, and CR18, are examined, it is obvious that the CR17.5 mix beats the others at both low and high loads.

This demonstrates that the CR17.5 has the best BSFC performance across a wide range of loads. When compared to other compression ratios, CR17.5 has lower BSFC ratings, indicating its capacity to achieve higher fuel efficiency and energy conversion. These findings highlight the significance of compression ratio selection in achieving efficient combustion and peak engine performance^36^. The preference for CR17 over CR16 and CR18 demonstrates that it strikes a favorable balance between compression efficiency and load requirements. This information contributes to the scientific study of engine performance characteristics and can aid in the development of strategies to increase fuel efficiency in compression ignition engines^9^.

**Figure 4:**
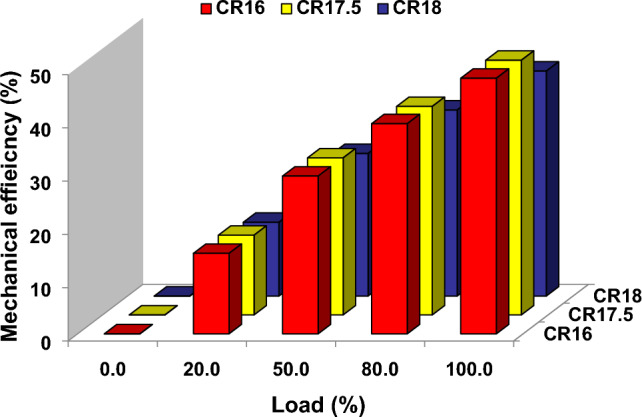
Load vs mechanical efficiency at different compression ratios.

Figure 4 demonstrates the relationship between load and mechanical efficiency while utilizing pure diesel fuel for various compression ratios (CR16, CR17.5, and CR18) at five distinct load levels (0, 20, 50, 80, and 100%). Mechanical efficiency is an important metric that quantifies the ratio of brake power generated at the crankshaft to the indicated work occurring within the combustion chamber of an engine. Analyzing the data reveals that mechanical efficiency levels vary depending on compression ratio and load conditions. Notably, when the compression ratios CR16, CR17.5, and CR18 are examined, CR17.5 consistently has the highest mechanical efficiency rating across all load levels. The importance of choosing the appropriate compression ratio in achieving the best engine performance and fuel efficiency cannot be overstated. According to data, CR17.5 offers the finest conditions for optimizing mechanical efficiency over the whole range of loads assessed. This conclusion highlights the importance of selecting an optimal compression ratio, as it directly affects the engine's overall performance and energy conversion efficiency^37^.

According to the data presented in Fig. 4, CR17.5 is the best combination of the compression ratios investigated (CR16, CR17.5, and CR18) in terms of mechanical efficiency, as it consistently produces the highest values across all tested loads. This information is useful for engine designers and researchers who wish to increase the efficiency and effectiveness of internal combustion engines by selecting the appropriate compression ratio. Similar trends are reported by other researchers^38^.

### Effect of blending ratios of biodiesel

**Figure 5:**
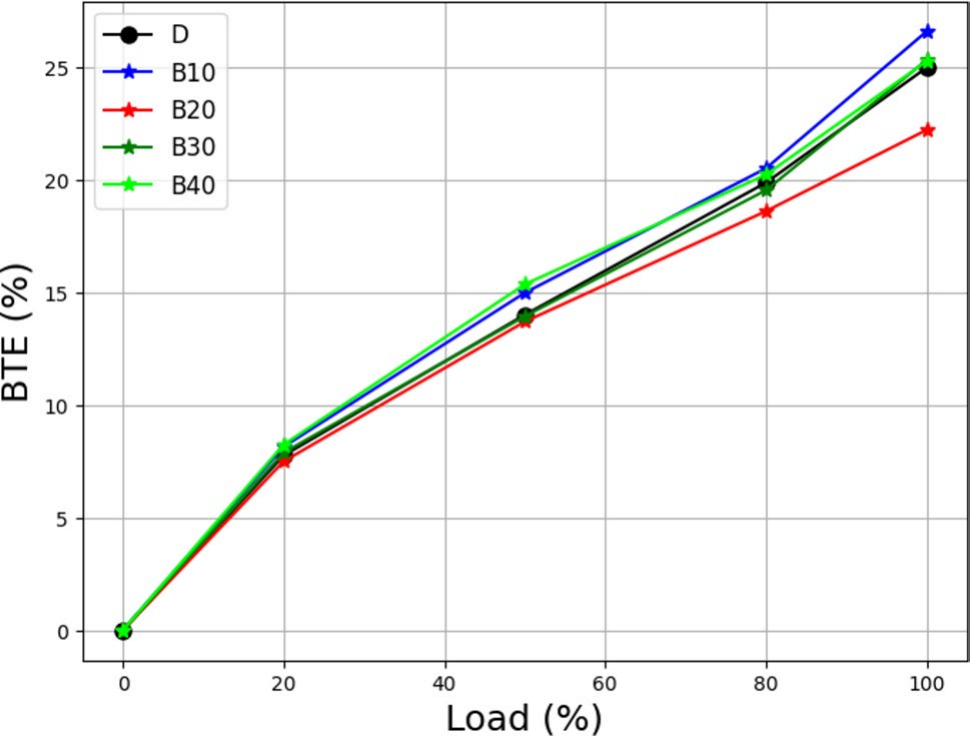
Load vs brake thermal efficiency at different biodiesel blend.

An engine's brake thermal efficiency (BTE) is a vital performance indicator that measures how efficiently fuel energy is transformed into usable effort. It denotes the link between the engine's output and the amount of fuel energy delivered. Figure 5 demonstrates the relationship between engine load and BTE for a specific Lemongrass oil biodiesel mix.

BTE is synonymous with fuel conversion efficiency and provides information about the engine's ability to convert fuel into mechanical work. In this study, the researchers investigated the BTE of the test engine employing a lemongrass oil biodiesel blend as the fuel source. The maximum and minimum BTE values for diesel were discovered to be 23.76 and 22.74%, respectively. Higher values indicate more efficient fuel usage. These values represent the engine's ability to convert the energy in the fuel into usable work^39^. The findings in Fig. 5 and associated BTE values illustrate the need to maximize fuel economy in internal combustion engines. Increasing BTE values is crucial for enhancing energy sustainability and lowering fuel use. This experiment provided valuable information about the performance parameters of the lemongrass oil biodiesel blend and its impact on engine efficiency^40^. These findings contribute to the body of knowledge in the field of alternative fuels and engine optimization, paving the way for the creation of more energy-efficient and environmentally friendly technology.

**Figure 6:**
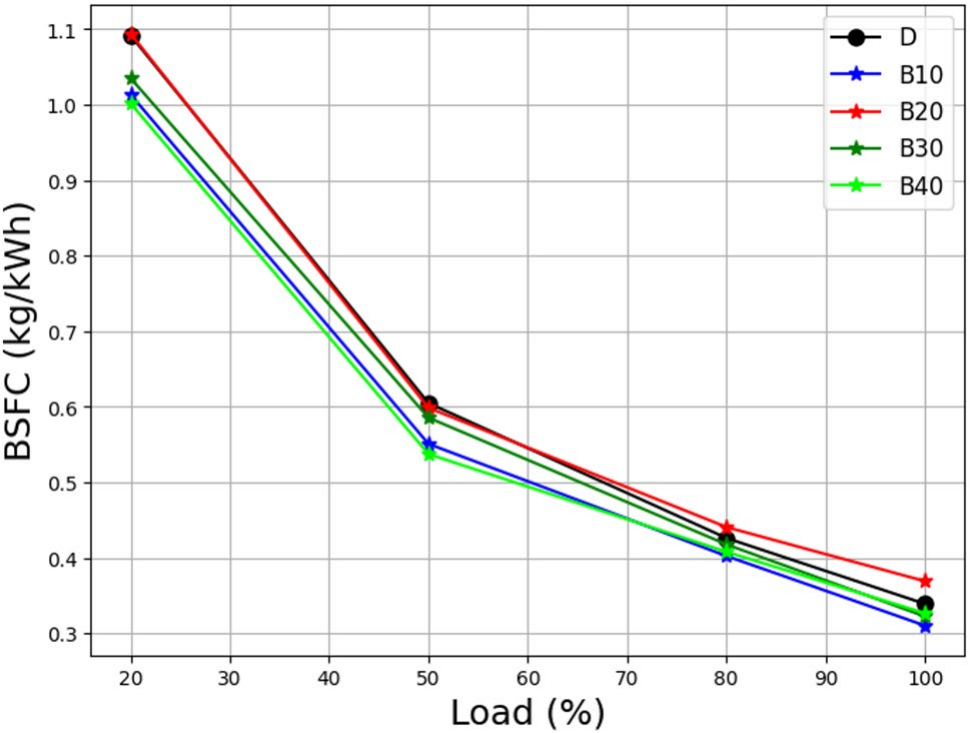
Load vs brake-specific fuel consumption at different biodiesel blends.

The concept of brake-specific energy consumption (BSEC) is an important criterion in evaluating the efficiency and performance of diesel engines. It is computed by multiplying the brake-specific fuel consumption (BSFC) by the fuel's calorific value. In Fig. 6 from the study "Load vs. Brake Specific Fuel Consumption of lemongrass oil biodiesel Blends," the relationship between load and BSFC for numerous lemongrass oil biodiesel blends is investigated.

The amount of energy needed to generate one unit of braking power is measured by BSEC, which provides information on the engine's overall efficiency. When employing BSEC as a metric, the evaluation of diesel engine capabilities becomes more precise because a variety of fuel blends with varying densities and calorific values are taken into account. Notably, the study discovers that the B30 blend has a higher BSFC than other mixes. This means that the engine consumes more fuel per unit of power produced in the B30 mix. The BSFC drops as the load increases, leading the BSEC to fall as well. This research reveals the engine's improved efficiency under high-load conditions^41^.

**Figure 7:**
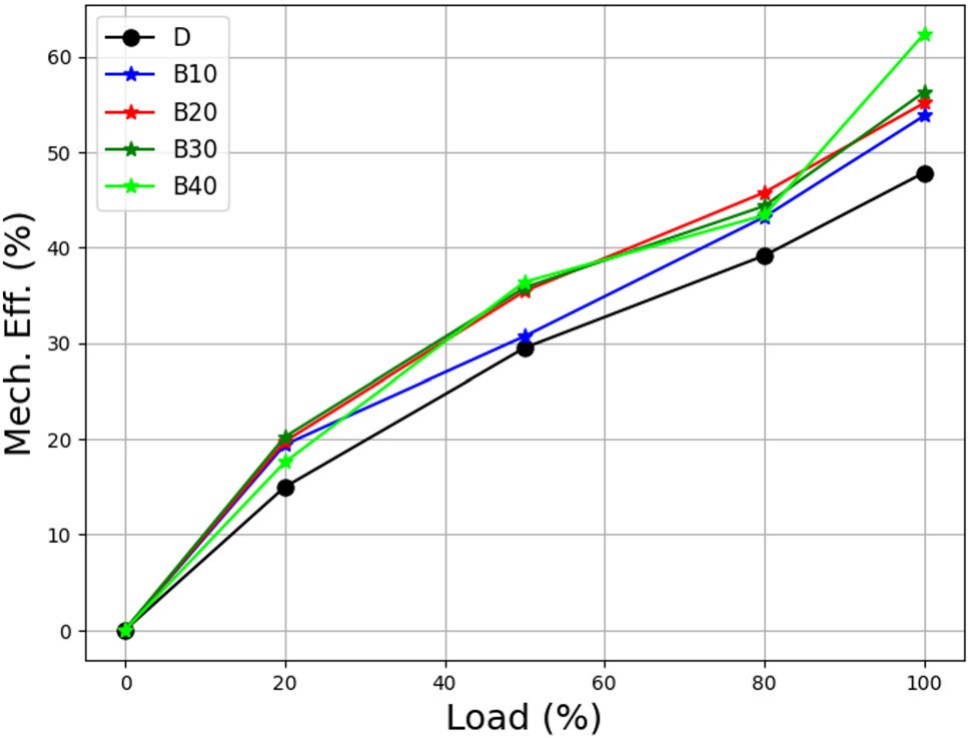
Load vs mechanical efficiency at different biodiesel blends.

The use of biodiesel blends in the study underscores the importance of understanding the impact of fuel composition on engine performance. The data demonstrates the potential advantages of biodiesel blends in terms of reduced fuel consumption and increased energy efficiency. It was also reported by other researchers^42^. The load vs. mechanical efficiency of the biodiesel blends of lemongrass oil is shown in Fig. 7, which demonstrates how mechanical efficiency rises with brake power. The B40 and B30 blends are more efficient than the remaining, almost identical diesel mixtures at higher loads. B10, B20, B30, and B40 are virtually as efficient as diesel at lighter loads. The percentages of diesel in B10 to B40 lemongrass oil biodiesel blends were 46.11%, 47.96%, 48.43%, and 49.09%, respectively.

**Figure 8:**
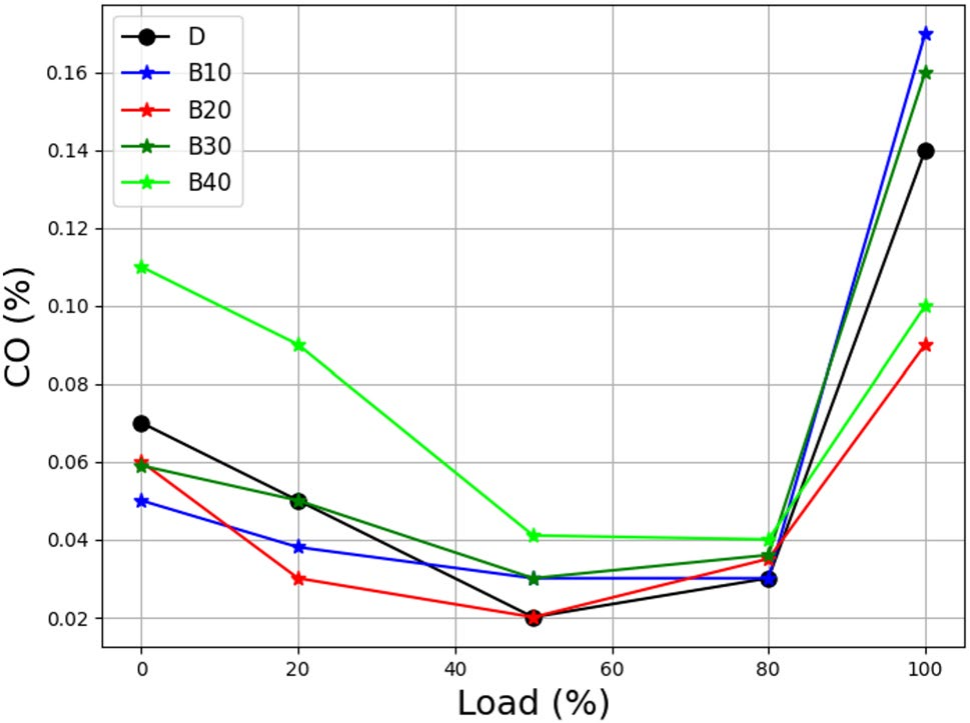
Load vs carbon monoxide at different biodiesel blends.

Figure 8 depicts the behavior of carbon monoxide (CO) emissions for several diesel blends (B10, B20, B30, and B40) under various engine loads. It has been noted that when the load increases, the CO emissions for B10 and B20 mixes stay high. This is due to inefficient fuel combustion, particularly when the engine operates at low loads and under air control, with the throttle just partially open^43^.

In contrast, both B30 and B40 blend significantly reduce CO emissions. This increase in combustion efficiency can be ascribed to an increase in engine load and a wider throttle. Increased engine load improves the combustion process for all mixes, resulting in less CO in the exhaust emissions. Surprisingly, for low-load mixes with low combustion efficiency, wide-open throttle has a large favorable effect on the fuel–air combination^44^. This design dramatically reduces CO emissions, indicating a more complete and efficient combustion process. These findings highlight the need to manage engine operating conditions such as load and throttle position to improve combustion efficiency and reduce CO emissions^45^. This knowledge can assist guide the development of ways for reducing emissions in diesel engines, ultimately contributing to the expansion of environmentally friendly and sustainable transportation systems. The results corroborate similar studies in this domain^46^.

**Figure 9:**
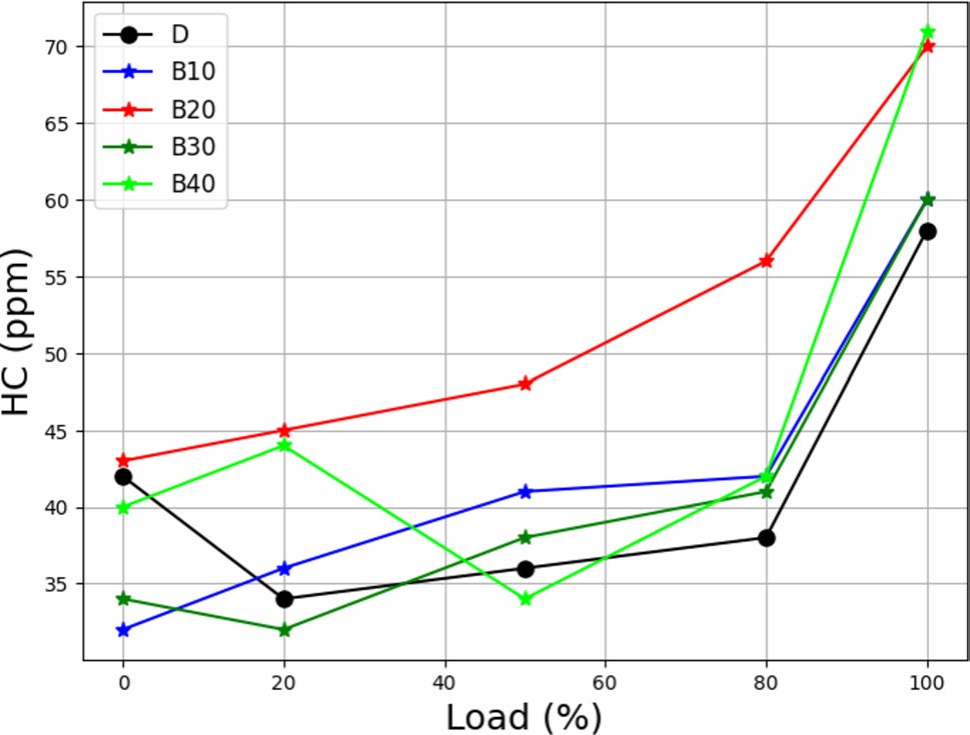
Load vs hydrocarbon emission at different biodiesel blends.

Figure 9 depicts the connection between loading and hydrocarbon (HC) emissions in lemongrass oil biodiesel mixes. According to the findings, when the load grows, lemongrass oil biodiesel blends' HC emissions exceed those of diesel. However, when the load increases, the HC emissions from B10 to B40 approach diesel emissions levels. At lower loads, lemongrass oil biodiesel blends perform nearly as well as diesel while producing fewer tailpipe pollutants. This problem is caused by an oxygen deficit in the engine cylinder caused by high loads^47^. Unburned hydrocarbons persist in the exhaust gases under these conditions, resulting in increased HC emissions. The behavior of all blends in terms of HC emissions closely resembles that of carbon monoxide (CO).

Both HC and CO are byproducts of combustion that is incomplete, that occur when there is more fuel in the combustion process than available oxygen. These results demonstrate the complex interplay of load, oxygen availability, and fuel concentration in influencing HC emissions in lemongrass oil biodiesel blends. Understanding these relationships is crucial for optimizing combustion processes, minimizing emissions, and encouraging the adoption of cleaner, more sustainable energy sources.

**Figure 10:**
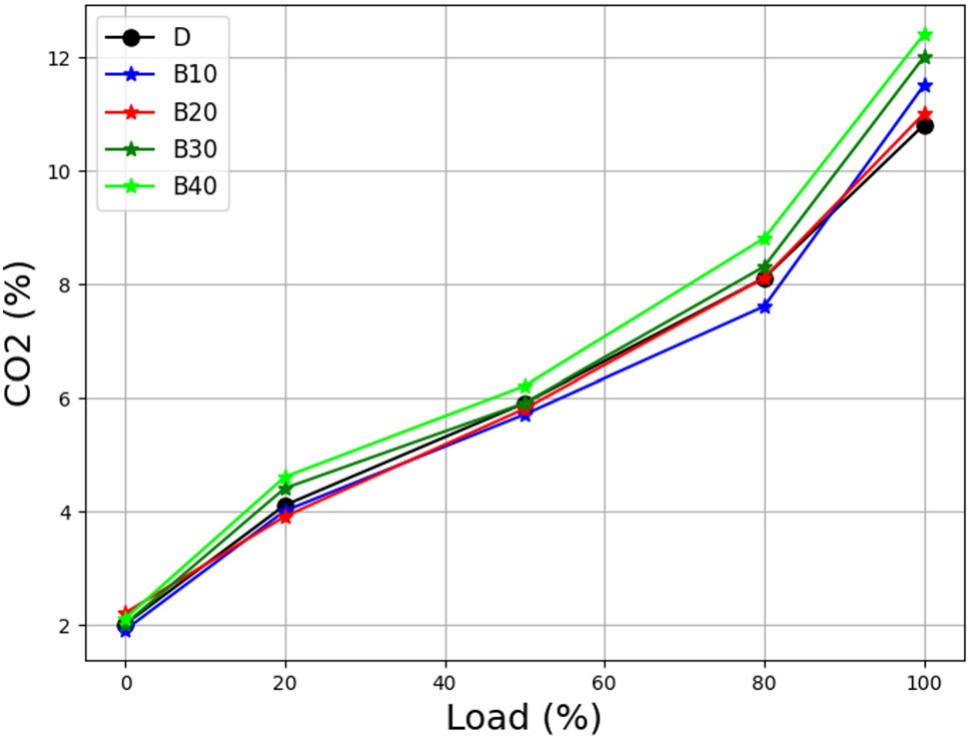
Load vs carbon dioxide at different biodiesel blends.

The load vs. CO_2_ emissions for lemongrass oil biodiesel blends are depicted in Fig. 10. At higher loads, B10 to B40 CO_2_ emissions are similar to CO_2_ emissions from pure diesel. At lower loads, all lemongrass oil biodiesel blends generate almost the same amount of CO_2_. Due to a poor combustion process brought on by a lack of oxygen in the air, CO is predicted to decrease with altitude rather than CO_2_. The results show that increasing CO_2_ emissions cause an oxygen shortage in the atmosphere^48^.

**Figure 11:**
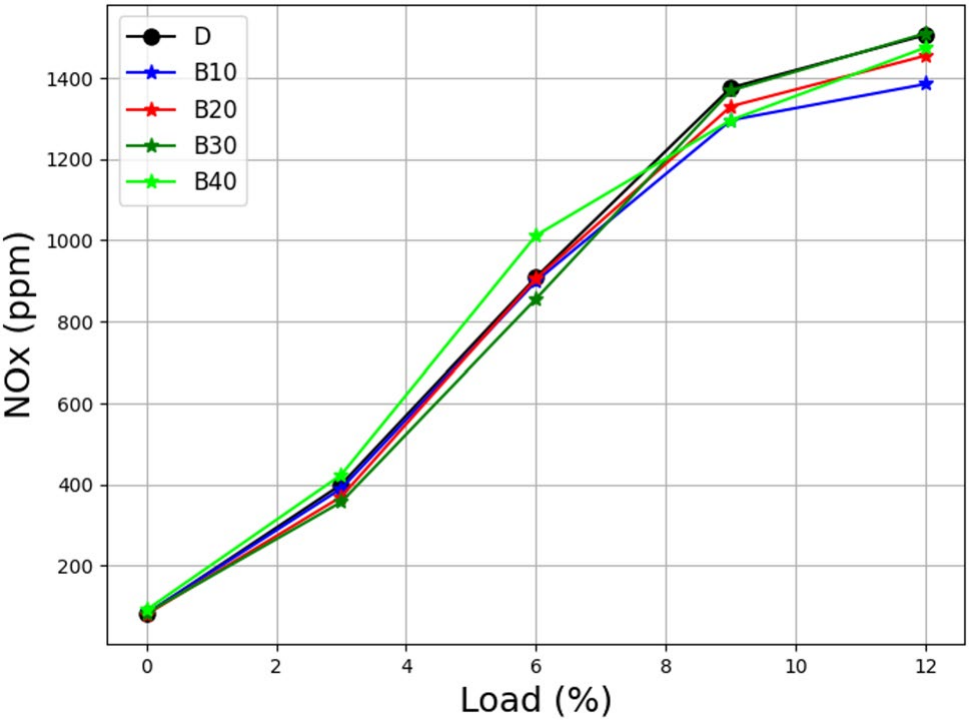
Load vs NOx at different biodiesel blends.

Figure 11 demonstrates the relationship between load and NOx emissions for several lemongrass oil biodiesel blends. Surprisingly, when the load increases, all blends create NOx emissions that are roughly comparable to diesel. At higher loads, however, B10 and B20 mixes generate less NOx than other lemongrass oil biodiesel blends. This finding implies that adopting these specific blends can effectively reduce NOx emissions.

NOx emissions levels are influenced by a number of parameters, including exhaust gas temperatures and the presence of oxygen in the combustion chamber. Blends with increased brake power may be more prone to producing excessive NOx levels as temperatures rise^49^. Higher temperatures promote more powerful reactions, which increase NOx production. However, combustion efficiency and the availability of air for reactions can counteract this trend. NOx creation can be minimized when combustion is efficient and there is enough air present, resulting in lower emissions. This emphasizes the need to improve combustion conditions and ensure adequate oxygen supply to reduce NOx formation^50^. From the above analysis, it could be found that B30 is the best selection to get high combustion efficiency, showing lower emission and higher performance than B10, B20, and B40. Thus, B30 is selected for the next experiments, in which B30 is blended with additives at a 2%, 4%, and 6% rate.

### Effect of blending ratios of additives

**Figure 12:**
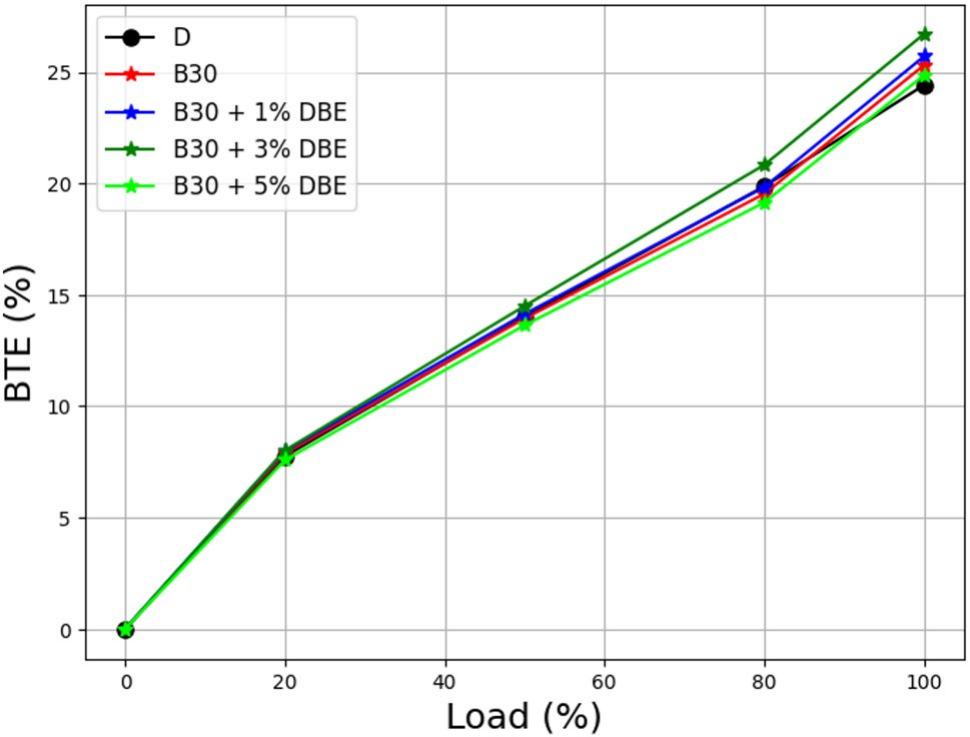
Load vs BTE at the different additive rate.

A comparison of the thermal efficiency of lemongrass oil biodiesel blends with regard to load and brakes is depicted in Fig. 12. Dibutyl ether is a component that is added to a biodiesel mixture at the additive level. In the process of combustion, the quantity of energy that is produced by a fuel and then converted into work that can be utilized is referred to as thermal efficiency. Because of the increased heat transfer to the cylinder wall, the BTE decreases in case of low engine speed. This is because of the temperature rise. When compared to B30 and its additions of 1%, 3%, and 5% dibutyl ether, the graph indicates that the biodiesel blend with the highest braking power efficiency is B30 + 3% DBE.

**Figure 13:**
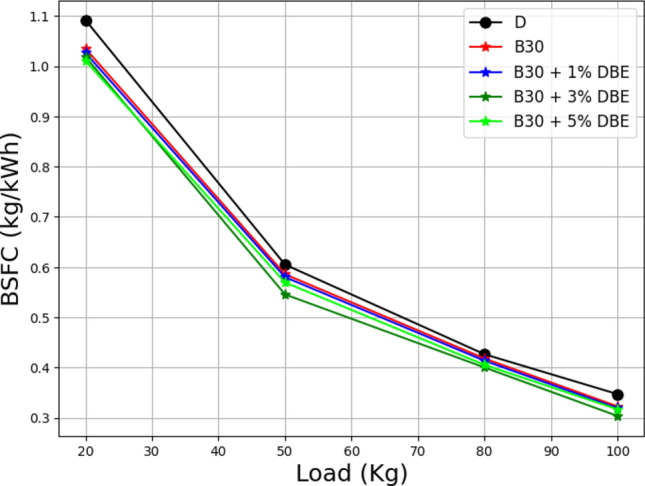
Load vs BSFC at the different additive rates.

The load vs. brake-specific fuel consumption of a lemongrass oil biodiesel blend with dibutyl ether is shown in Fig. 13. The effect of engine speeds at full load on the BSFC for lemongrass oil biodiesel mixes. Low engine speed leads to inefficient combustion and a strong quenching effect, which increases the BSFC. BSFC decreases as engine speed rises, combustion improves, and overall energy consumption increases. The density, viscosity, and heating valves create the BSFC. According to the graph, B30 + 4% DBE is the biodiesel mix with the best brake power efficiency among B30 and its additives of 2%, 4%, and 6% dibutyl ether^51^.

**Figure 14:**
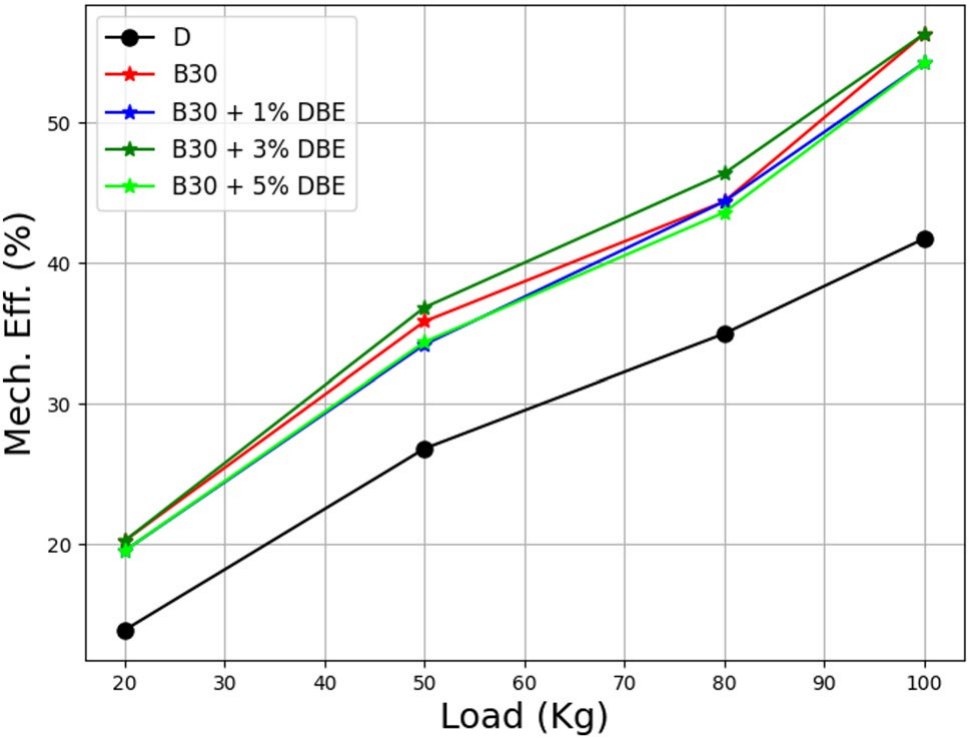
Load vs mechanical efficiency at the different additive rate.

Figure 14 shows the relationship between load and mechanical efficiency for a B30 lemongrass oil biodiesel mix with various additives at 2%, 4%, and 6% dibutyl ether. The evaluation of a mechanical system's performance efficacy. It is typically the ratio of the mechanical system's output to input power, and because of friction, this efficiency is less than one.

## Conclusion

This research examines the efficiency and emissions of a diesel engine that runs on lemongrass oil biodiesel-diesel blends with dibutyl ether added at 1%, 3%, and 5% rates. The results of the lemongrass oil biodiesel blend are compared to those of diesel. The following are the main observations:

- Blends of biodiesel have the same physical characteristics as diesel. A diesel engine that has not been modified in any manner is used to evaluate the prepared fuel samples.
- The CR17.5 is the best of the other two diesel compression ratios, which are all different. Because performance gains are greatest and emissions are least with higher loads
- Diesel engines can be powered with blends of lemongrass oil biodiesel without modification. Citronella oil gives the engine a smooth, diesel-like performance.
- In comparison to diesel, brake thermal efficiency is worse at higher loads. Lower loads lead to lower specific fuel consumption. In the B30 blend, mechanical efficiency at higher loads is at its highest, while emission metrics including CO, CO_2_, HC, and NOx were lowered with the introduction of an additive, although HC is increased with greater loads of lemongrass oil biodiesel blends.
- The B30 + 4% additive achieves maximum efficiency at the fourth load in terms of both brake power and mechanical efficiency when compared to the B30 biodiesel blend with various composition additives.

Future studies should focus on optimizing additive concentrations, exploring long-term engine durability, and assessing the economic and environmental impacts of large-scale lemongrass oil biodiesel production and use.

## Data Availability

The data is available within the manuscript.

## Abbreviations

B10: 90% diesel + 10% biodiesel
B20: 80% diesel + 20% biodiesel
B30: 70% diesel + 30% biodiesel
B40: 60% diesel + 40% biodiesel
CR: Compression ratio
CRDi: Common rail diesel injection
VCR: Variable compression engine
BSFC: Brake specific fuel consumption
CNT: Carbon nanotube
PBDF: Petroleum-based diesel fuel
SFC: Specific fuel consumption
kW: Kilowatt
UHC: Unburnt hydrocarbon
DBE: Dibutyl ether
BSEC: Brake specific energy consumption
BTE: Brake thermal efficiency
NOx: Oxides of nitrogen
CO: Carbon mono oxide
CO_2_: Carbon dioxide
ER: Equivalence ratio
MAPE: Mean absolute percentage error
MSE: Mean squared error
GWO: Grey wolf optimizer
SDG: Sustainable development goal
HC: Hydrocarbon
